# Origami-/kirigami-inspired structures: from fundamentals to applications

**DOI:** 10.1098/rsta.2024.0018

**Published:** 2024-10-07

**Authors:** Suyi Li, Mohammed F. Daqaq

**Affiliations:** ^1^ Virginia Tech, Blacksburg, VA, USA; ^2^ Department of Mechanical Engineering, New York University, Abu Dhabi, Saadiyat Island, UAE

**Keywords:** origami, kirigami

## 1. Introduction

Over the past two decades, we have witnessed the rapid emergence of origami and kirigami as versatile design and fabrication platforms for incubating engineering innovations. This themed issue aims to exemplify this trend by showcasing some of the most cutting-edge research in this vibrant field.

The original ideas of origami (‘fold-paper’ in Japanese) and kirigami (‘cut-paper’) were simple and elegant: one can cut, fold and manipulate a piece of paper into a decorative three-dimensional (3D) shape, like a paper crane or pop-up book. These folk arts originated from East Asia centuries ago. Their design and complexity have remained the same throughout this long history. The first breakthrough occurred after World War II, as artists and mathematicians developed new folding techniques and mathematical theories for designing and analysing cutting and folding. As a result, we have witnessed an explosion in the beauty and complexity of these ancient arts. The seemingly infinite possibility of creating 3D shapes out of two-dimensional (2D) flat precursors quickly attracted attention from scientists and engineers, who turned origami and kirigami into a broad research discipline.

The first breakthrough in origami and kirigami research occurred in design and folding kinematics, it is not surprising, therefore, that early engineering adoptions also focused on exploiting shape transformations via folding and cutting. The actions of folding and cutting divide a 2D sheet into a tessellation of rigid facets; in this way, origami and kirigami sheets behave like 3D linkage mechanisms. Deployable solar panels for spacecraft, kinetic building facades and paper packaging are good examples of origami and kirigami kinematics put to use.

Since around 2010, we have witnessed a paradigm shift in origami and kirigami research. The focus has moved away from the pure kinematics and into the mechanics induced by folding and cutting. This shift has led to a new way of analysing origami and kirigami, treating them more like elastic membranes or shells with folds and cuts, rather than linkages. With such a paradigm shift, the breadth and depth of origami/kirigami-inspired engineering study has quickly reached a new level.

Three key driving factors have enabled this shift in research area. The first is the development of new mechanics and dynamics models. These models harvest elastic energy from the folding along crease lines and complex facet deformations via bending and stretching. Inertial effects can also be incorporated to reveal the unique and nonlinear dynamical behaviours in origami and kirigami structures. For example, the article by Anandaroop and Pratapa in this themed issue aimed to improve the bar-hinge model, one of the most popular reduced-order models for origami. The article by Fang *et al*. assessed the fidelity of different dynamic modelling approaches for multi-stable origami. The article by Nassar *et al.* explored the fundamental mechanics behind the bending of periodic and folded surfaces.

The second driving factor is the exploration of new cutting and folding designs. There are many notable attempts to generalize the classical origami and kirigami designs to enable new performance space. Moreover, one can assemble multiple origami or kirigami sheets into a space-filling 3D topology. For example, the article by Deshpande *et al*. generalized the design of Yoshimura origami to create massively reconfigurable and deployable booms. The article by Grasinge *et al*. systematically examined the kinematics of higher-degree vertices. The article by Li *et al*. explored varying mountain and valley assignments of a doubly corrugated origami pattern to program its mechanical properties and folding behaviour. The article by Almessabi *et al*. studied the effect of panel confinement on the possible equilibria, mechanical properties and energy absorption characteristics of a class of origami-inspired metamaterials.

The third factor is material innovation; cutting and folding principles can apply to thin precursors at vastly different sizes, from microscale graphene sheets and mesoscale electronics to macroscale metals and polymers. Moreover, thin and flat precursors make it easy to embed other functional components. As a result, one can leverage these functional materials to enrich the overall mechanics and functionality. For example, the article by Lerner *et al*. described how to integrate heat-responsive shape memory polymers into origami to create reconfigurable folding structures. The article by Pruett *et al*. proposed magnetically stabilized hinges to actuate and deploy an origami-inspired mechanism.

As a result of these developments, we see broad applications of origami and kirigami in many different fields. In this regard, many articles in this themed issue reflect the vibrant possibilities that origami and kirigami principles can offer. For example, the article by Li *et al*. explored complex morphing mechanical metamaterials using a hybrid kirigami–origami design. The article by Khazaaleh *et al*. presented energy-harvesting floor tiles with Kresling origami inserts. The article by Hwang *et al*. examined highly effective adhesives with hierarchical kirigami cuts. The article by Jiao *et al*. presented a multi-stable kirigami material system that can achieve mechanical proprioception. The article by Yao *et al*. used the flasher pattern to design a foldable origami antenna. The article by Tomohiro and Sunao demonstrated that metamaterials with adaptively tunable bandgaps and wave coupling can be created by periodically connecting rotated and unrotated Miura Ori tubes.

Despite the extensive studies into origami and kirigami, the potential for new discoveries in this field remains vast. Here, we briefly highlight four exciting new topics:

— Exploration of new designs: the design space of origami and kirigami is far from exhausted. We can significantly expand the design by strategically combining cutting and folding, introducing curved creases and creating hierarchical patterns. Establishing mappings between new origami/kirigami designs and their mechanical properties—either by first-principle modelling or data-driven approaches—remains a critical and open question.
— Multi-modal deformation: traditionally, an origami or kirigami pattern is designed for only one deformation pattern; however, there are exciting possibilities of multiple deformation modes by selectively engaging different groups of creases. For example, selectively folding different groups of creases in a hybrid origami–kirigami module can result in topologically unique and stiff states, creating metamaterials with switchable mechanical behaviours.
— Cross-discipline functionality: the ongoing convergence of advanced manufacturing, computing science and biology has expanded the scope of origami/kirigami studies beyond traditional engineering boundaries. For example, multi-stable material ‘bits’ inspired by origami and kirigami designs have become the foundation of mechanical computing and physical intelligence. Origami and kirigami can also serve as scaffolds from which new living materials might be engineered.
— Fabrication tools and materials: real-life implementation of origami-inspired structures cannot be achieved without fabrication methods that combine automation, repeatability and low cost with new materials that are robust, durable and can withstand the elements.

The beauty and elegance of origami and kirigami have attracted the attention of researchers from many different disciplines. Future success in this field will continue to hinge upon the synergy of broad research collaborations. In addition, discovering new functionalities will continue to energize this exciting area of research.

## Data Availability

This article has no additional data.

